# The relationship between disc morphology and condylar bone marrow edema in adolescent patients with anterior disc displacement with reduction: a retrospective cross-sectional study

**DOI:** 10.1186/s12903-026-08827-0

**Published:** 2026-06-12

**Authors:** WenZhu Dang, Zhuo Wang, YanLi Jiang, YuRong Ma, FengXian Fan, Jing Zhang

**Affiliations:** 1 Department of Magnetic Resonance, The Second Hospital & Clinical Medical School, Lanzhou University, No. 82 Cuiyingmen, Chengguan District, Lanzhou, 730030 China; 2 Gansu Province Clinical Research Center for Functional and Molecular Imaging, Lanzhou, China

**Keywords:** Temporomandibular joint disorders, Anterior disc displacement with reduction, Adolescents, Magnetic resonance imaging, Bone marrow edema, Disc morphology

## Abstract

**Background:**

Temporomandibular disorders (TMD) are relatively common among adolescents, with anterior disc displacement with reduction (ADDwR) being the most prevalent subtype. Current research has primarily focused on assessing disc position; however, the morphological characteristics of the disc in adolescent ADDwR patients and their association with early degenerative changes in the condyle, such as bone marrow edema (BME), remain underexplored. Therefore, this study aims to utilize 3.0T MRI to compare disc morphology and condylar bone marrow signals between adolescent ADDwR patients and a comparison group, as well as to further investigate the potential relationship between disc morphology and early condylar bone alterations.

**Methods:**

We conducted a retrospective cross-sectional study on 84 participants: 44 in the ADDwR group and 40 in the comparison group without TMD (headache patients with no clinical or MRI signs of TMD). Independent two-sample t-tests, Mann-Whitney U tests, chi-square tests, or Fisher’s exact tests were used for intergroup comparisons. The association between posterior band thickness and BME was assessed using the Mann-Whitney U test (with rank-biserial correlation as effect size). Firth logistic regression was used to identify factors independently associated with BME.

**Results:**

The ADDwR and comparison groups were comparable in age and sex distribution (both *p* > 0.05). The ADDwR group showed more frequent disc deformation, thicker posterior band, and higher occurrence of condylar BME and joint effusion than the comparison group (all *p* < 0.001). In the ADDwR group, patients with BME had greater posterior band thickness than those without BME (median 3.70 mm vs. 3.20 mm; Mann-Whitney U = 276.5, *p* < 0.001; rank-biserial correlation *r* = 0.44). In multivariable Firth logistic regression adjusting for age, sex, disc deformation, and joint effusion, posterior band thickness was the only factor independently associated with condylar BME (OR per 0.1 mm increase = 1.64, 95% CI: 1.16–2.32, *p* = 0.0049). None of the other covariates reached statistical significance.

**Conclusion:**

Adolescent ADDwR patients present with abnormalities in both articular disc morphology and condylar bone marrow signals. Posterior band thickening is independently associated with condylar BME. These cross-sectional findings are hypothesis-generating; prospective studies are needed to determine whether posterior band measurement could help identify patients at risk of developing BME.

**Supplementary Information:**

The online version contains supplementary material available at 10.1186/s12903-026-08827-0.

## Background

Temporomandibular disorders (TMD) are common orofacial pain conditions associated with pain, functional limitations and joint clicking [1]. Adolescents are a high-risk group for TMD owing to the combined effects of mandibular growth and development, hormone fluctuations and psychological stress [2]. Still, the majority of TMD work has been conducted in adults and the specific characteristics of internal derangement in adolescents are not well known.

Anterior disc displacement with reduction (ADDwR) is the most common subtype of disc displacement in adolescent population. Clinically, joint clicking is a common screening sign for ADDwR. However, it has been shown from MRI studies that clicking by itself does not reliably predict disc position [3]. Hence, MRI confirmation is necessary for accurate diagnosis. The initial screening clue we used in this study was clinical clicking; however, final inclusion was based on MRI confirmation. Moreover, MRI allows for an accurate assessment of disc morphology, especially posterior band thickness, which is the main variable of this study.

Bone marrow edema (BME) in the adolescent condyle is a key early change that often gets overlooked. It is believed to be a reversible change and can occur before the clinical onset of osteoarthritis (OA). In adults, BME is usually considered a late-stage finding [4]. In contrast, adolescents have condyles that are still maturing—subchondral cortical bone formation goes on throughout the teenage years [5]. This continuing development may render the condyle more sensitive to the biomechanical shifts associated with ADDwR.

Yet most of the existing evidence comes from adults or concerns disc position rather than morphology. There has not been a quantitative study examining disc morphology and condylar BME in adolescent patients with ADDwR. A lack of knowledge limits our understanding of early bony changes in this group.

Based on this, we propose the following hypothesis: In adolescents with ADDwR, altered disc morphology, specifically posterior band thickening, is independently associated with condylar BME. This hypothesis hinges on a plausible biomechanical pathway, where a thickened posterior band would pass on aberrant loading to the condyle. The current study applied a cross-sectional design to test the hypothesis.

The study aimed to compare disc morphological parameters between adolescents with ADDwR and a comparison group (patients presenting with headache, but free of TMD signs on both clinical and MRI examinations), to evaluate the association between quantitative disc parameters and condylar BME, and to provide some evidence to help future prospective studies on early intervention strategies.

## Methods

### Study design and participants

#### Participant inclusion and grouping

This retrospective cross-sectional study enrolled adolescents aged 12–18 years who underwent temporomandibular joint (TMJ) MRI at the Second Hospital of Lanzhou University from January 2024 to October 2025. All subjects received standardized clinical exams according to the Diagnostic Criteria for Temporomandibular Disorders (DC/TMD) Axis I, done independently by two trained oral and maxillofacial surgeons. The ADDwR group consisted of 44 patients and the comparison group included 40. The groups were frequency-matched for age (± 1 year) and sex. The t-test and chi-square test were used to measure balance, and standardized mean differences (SMD) were calculated for age (0.19) and sex (0.22), which suggest the two groups were reasonably comparable. Age and sex were also controlled for in the multivariable regression models.

It is noteworthy that, although the presence of clinical clicking does not strictly correspond to MRI disc position [3], reproducible clicking remains an essential clinical characteristic of ADDwR based on DC/TMD criteria. In the present study, clicking was used as the initial screening clue while the final diagnosis was MRI-confirmed ADDwR. This method enhances the diagnostic specificity of MRI, though it is compliant with the internationally accepted clinical diagnostic framework.

#### Inclusion criteria for the patient group

Patients were required to meet both of the following:

1. Reproducible joint clicking on clinical examination according to DC/TMD Axis I (on opening or closing, confirmed by auscultation or palpation).
2. MRI findings showing anterior disc displacement on closed‑mouth position with reduction to normal disc‑condyle relationship on open‑mouth position (i.e., ADDwR).

#### Inclusion criteria for the comparison group

The comparison group consisted of adolescents who underwent TMJ MRI for headache evaluation during the same period to rule out TMD, and who met all of the following:

1. No TMD diagnosis according to DC/TMD Axis I (including absence of joint clicking, pain on mandibular movement, limited mouth opening, and muscle tenderness).
2. Normal bilateral TMJ on MRI (no disc displacement, no disc morphological abnormalities, no condylar BME).

#### Exclusion criteria (applicable to both groups)

For both groups, the exclusion criteria included previous TMJ trauma or surgery, systemic inflammatory arthritis, congenital craniofacial syndromes, and an incomplete MRI or images of insufficient quality for diagnosis.

#### Selection of one joint per patient

To meet the assumption of statistical independence, the principal analysis was performed at the patient level with only one TMJ per participant. For patients with bilateral ADDwR, the selected side was the worse or more symptomatic side based on clicking loudness or presence of pain. The affected side was chosen for patients with unilateral ADDwR. For the comparison group of headache patients without TMD, one side was randomly chosen. By concentrating on the clinically relevant joint, this approach respects the independence of observation.

### Clinical data collection

Clinical data for this study were collected through a retrospective review of electronic medical records. The presence or absence of joint sounds and their laterality were recorded. Visual analog scale (VAS) scores of pain intensity were extracted from the case records. Patients self-reported the VAS score, rated from 0 to 10, where 0 means no pain and 10 means the worst pain imaginable. The VAS score measures the maximum intensity in the joint or masticatory muscles at assessment. The maximum active mouth opening was defined as the maximum vertical distance (in millimetre) between the incisal edges of the upper and lower central incisor when the patient was asked to open his mouth as wide as possible without any pain.

### MRI scanning protocol and image analysis and diagnostic criteria

#### Scanning protocol

All TMJ MRI examinations were performed on a 3.0T Philips Ingenia scanner using a dedicated 16-channel head-neck coil. For each subject, the oblique sagittal and oblique coronal imaging planes were aligned parallel and perpendicular to the long axis of the mandibular condyle, respectively. The ensuing sequences were obtained.

1. Oblique sagittal T1-weighted (T1WI) turbo spin echo (TSE)

Parameters: TR/TE=900/20 ms, slice thickness=3.0 mm, matrix size=228×227, field of view=160×160 mm, number of slices=28.

(2) Oblique sagittal proton density-weighted (PDW) TSE with fat suppression

Parameters: TR/TE = 2000/21 ms, slice thickness = 3.0 mm, matrix size = 256 × 205, field of view = 160 × 160 mm, number of slices = 28.

(3)  Oblique sagittal T2-weighted (T2WI) TSE with fat suppression and motion compensation

Parameters: TR/TE = 1969/90 ms, slice thickness = 3.0 mm, matrix size = 320 × 320, field of view = 160 × 160 mm, number of slices = 28.

(4)  Oblique sagittal dynamic single-shot T2WI cine imaging

Parameters: TR/TE = 3000/130 ms, slice thickness = 5.0 mm, matrix size = 368 × 259, field of view = 220 × 194 mm, number of slices = 5, temporal resolution ≈ 3 s/frame.

(5)  Oblique coronal PDW TSE with fat suppression

Parameters: TR/TE = 2000/21 ms, slice thickness = 2.0 mm, matrix size = 320 × 240, field of view = 160 × 120 mm, number of slices = 40.

(6)  Oblique coronal T2WI TSE with fat suppression

Parameters: TR/TE = 2000/90 ms, slice thickness = 3.0 mm, matrix size = 300 × 220, field of view = 150 × 110 mm, number of slices = 42.

For the phase-encoding direction, all the sequences employed a parallel imaging (SENSE) acceleration factor of 2. After acquisition, the pictures were sent to PACS and evaluated by two radiologists who were unaware of the clinical details.

#### Image analysis and diagnostic criteria

Two radiologists with at least 5 years of experience in the diagnosis of MRI of the head and neck, blinded to clinical details, independently evaluated all MRI images. Before the formal evaluation, the two radiologists underwent a calibration session using 20 randomly selected TMJ MRI scans (not included in the study). Any discrepancies were resolved by discussion. The imaging evaluation criteria for disorders in the TMJ mentioned here are internationally recognized [6]. This study is a cross-sectional study with a limited sample, and to avoid excessive subdivision leading to insufficient number of cases, less statistical power, and inter-observer variability, a conservative binary classification method focusing on definitive pathological changes was undertaken.

- Disc Position Assessment: Evaluated on oblique sagittal PDW images in the closed-mouth position.
  Normal: The posterior band of the disc lies above the condylar apex, which is seen in the sagittal plane between the 11 o’clock to 1 o’clock position.
  ADDwR: The disc is anteriorly displaced when closing the mouth, with the posterior band of the displaced disc located anterior to the apex of the condyle which is typically at the 10 o’clock position or more anterior. The cine sequences show that the disc moves to a normal or near-normal position on mouth opening.
- Disc morphology: Evaluated on oblique sagittal PDW images.
  Normal: The disc exhibits a biconcave shape (“bow-tie” appearance) with homogeneous signal intensity.
  Deformation: Includes any recognizable disruptions of a morphologic type as well as loss of biconvex shape (biconvex, folding, thickening, or thinning).
- Posterior band thickness measurement
  Posterior band thickness was measured on oblique sagittal T2‑weighted images (fat‑suppressed, TR/TE = 1969/90 ms) with the mouth in the closed‑mouth position. The slice showing the best visualisation of the disc and condyle was selected (typically the central portion of the condyle). On that slice, the thickness of the posterior band was defined as the maximum distance from its superior to inferior border, measured perpendicular to the long axis of the band using PACS‑integrated callipers (precision 0.01 mm). For each TMJ, three independent measurements were taken, and the average value was used for analysis. In addition, a schematic diagram illustrating the measurement method is provided as Fig. S1 (Supplementary Material). To ensure reproducibility, the two radiologists independently measured posterior band thickness in all participants. The intra‑rater ICC (assessed by re‑measuring 20 randomly selected scans after a two‑week interval) was 0.96 (95% CI: 0.90–0.98), and the inter‑rater ICC was 0.95 (95% CI: 0.91–0.97).
- The assessment of marrow signal in the condyle was made by comparing oblique sagittal T1WI with T2WI (T2_SPAIR).
  Normal: The signal intensity is isointense to the surrounding normal cancellous bone across both sequences.
  Edema: On fat-suppressed T2WI, there is a poorly marginated area of high signal intensity within the condylar marrow, which appears isointense or slightly hypointense on corresponding T1WI.
- Joint effusion: Joint effusion was evaluated using oblique sagittal fat-suppressed T2WI (T2_SPAIR) images.
  Absent: There is no appreciable fluid signal in the upper and lower joint spaces.
  Present: A linear, striated or patchy area with a well-defined high signal intensity is seen in the joint.
  Each of these indicators was evaluated independently by two physicians. The final data were subject to statistical analysis according to the consensus reached by consultations.

### Statistical analyses

All statistical analyses were performed using SPSS version 27.0 and R version 4.5.2. This was an exploratory retrospective study; therefore, no formal sample size calculation was performed a priori. The results should be interpreted as hypothesis‑generating and require validation in larger cohorts. Given the exploratory nature of this study, no correction for multiple comparisons was made; therefore, the results should be interpreted conservatively.

Normality of continuous variables was assessed using the Shapiro-Wilk test. Normally distributed data are presented as mean ± standard deviation (SD), and non‑normally distributed data as median with interquartile range (IQR). Categorical variables are presented as frequencies and percentages.

Intergroup comparisons were performed using the independent two-sample t-test (for normal continuous variables), Mann-Whitney U test (for non-normal continuous variables), and Pearson’s chi-square test (for categorical variables). Fisher’s exact test was used when any expected cell count was less than 5.

The analysis was done using the Mann-Whitney U test to check posterior band thickness in patients with and without condylar BME. Effect size calculation was done using rank-biserial correlation. The distribution of the data was visualized using violin plots.

Due to complete separation (zero events in the control group for BME and disc deformation), standard logistic regression was not feasible; therefore, Firth penalized logistic regression was applied. Posterior band thickness was scaled per 0.1 mm to improve interpretability, as absolute differences between groups were typically less than 1 mm. A Firth penalized logistic regression model was fitted with posterior band thickness (per 0.1 mm), age (per year), sex, disc morphology (deformed vs. normal), and joint effusion (present vs. absent) as covariates to identify factors independently associated with condylar BME. Standard demographic confounders included age and sex. Disc morphology and joint effusion were selected because they are commonly found on MRI and may influence BME. Other clinical factors (e.g., duration of symptoms, bruxism, trauma, occlusion) were not collated systematically in this retrospective study (see Limitations). The ratio of events to variables is roughly 2.4, but for a statistical analysis of this kind, the ratio needs to be at least 10. Thus, the analysis is exploratory, as detailed in the article. A likelihood ratio test was used for assessing model fit.

A two-sided *p*-value < 0.05 was considered statistically significant.

## Results

### Demographic and clinical characteristics

A total of 84 participants were enrolled. The ADDwR group included 44 participants, and the comparison group included 40 participants. As shown in Table 1, there were no significant differences between the two groups in age, sex, or maximum mouth opening (all *p* > 0.05), indicating good comparability. Clicking side and VAS scores were recorded for the ADDwR group only, as these were not applicable to the comparison group.

**Table 1:** Demographic and Clinical Characteristics of Participants with ADDwR and Comparison Group

Characteristic	ADDwR Group (*N* = 44)	Comparison Group (*N* = 40)	*P*	SMD
Age (years)	15.48 ± 1.69	15.15 ± 1.76	0.388^#^	0.19
Gender, n (%)			0.348^*^	0.22
Male	11 (25.0%)	14 (35.0%)		
Female	33 (75.0%)	26 (65.0%)		
Maximum mouth opening (mm)	41.11 ± 4.78	41.63 ± 3.54	0.582^#^	0.12
Clicking Side, n (%)			NA	NA
Left	21 (47.7%)	-		
Right	23 (52.3%)	-		
VAS Score for TMJ discomfort (0–10)	5.0 (4.0, 6.75)	-	NA	NA

Note: Values are mean ± SD unless otherwise indicated. VAS is presented as median (IQR). ^#^Independent two sample t-test; ^*^Chi-square test. SMD = standardized mean difference (for continuous variables, SMD equals Cohen’s d; for sex, Cohen‘s h). *VAS* visual analogue scale, *TMJ* temporomandibular joint; -: not applicable

### Descriptive imaging findings

**Fig. 1:**
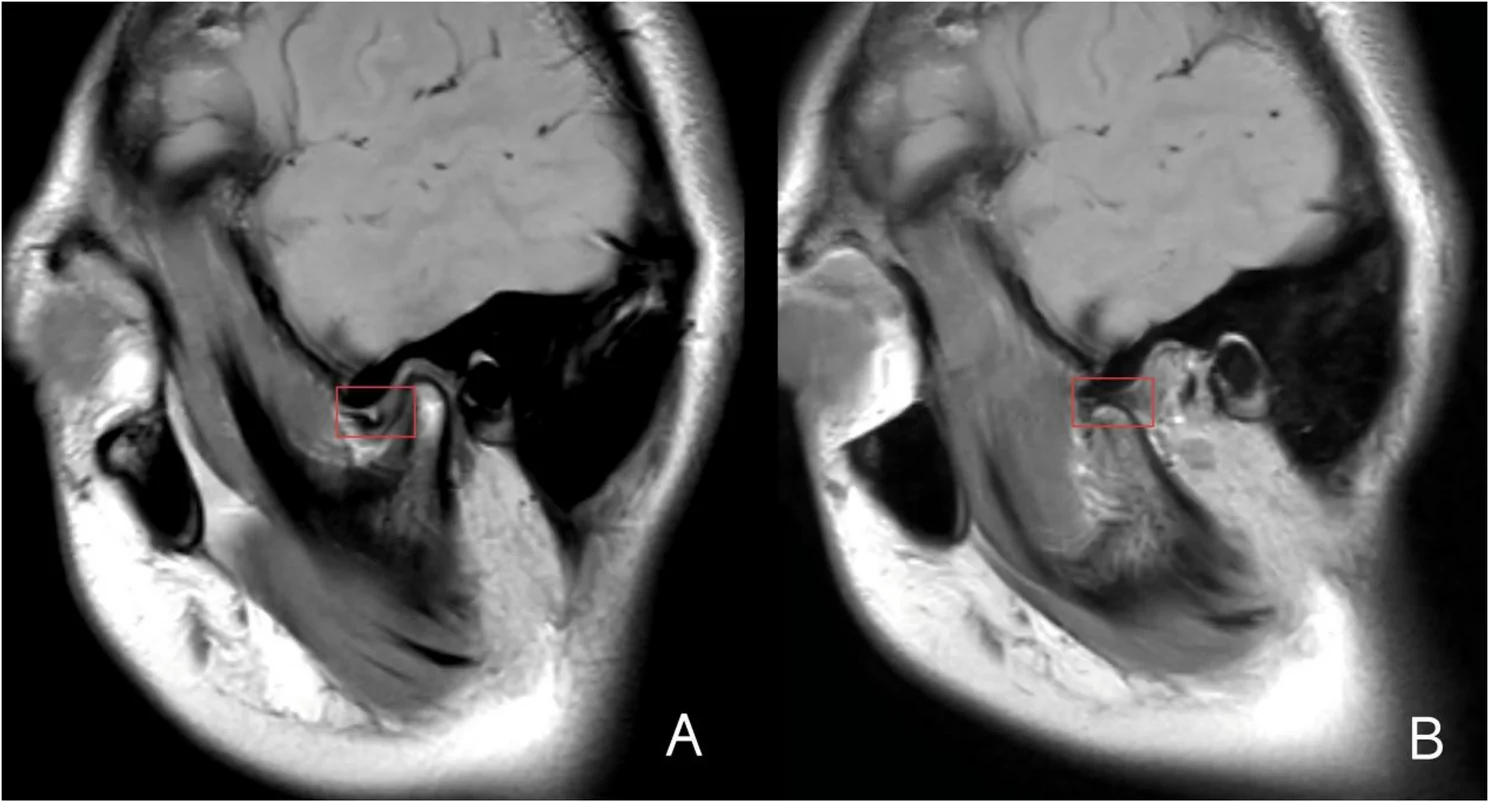
Representative MRI of an ADDwR patient: (**A**) closed-mouth position showing anterior disc displacement (red box); (**B**) open-mouth position showing disc reduction

**Fig. 2:**
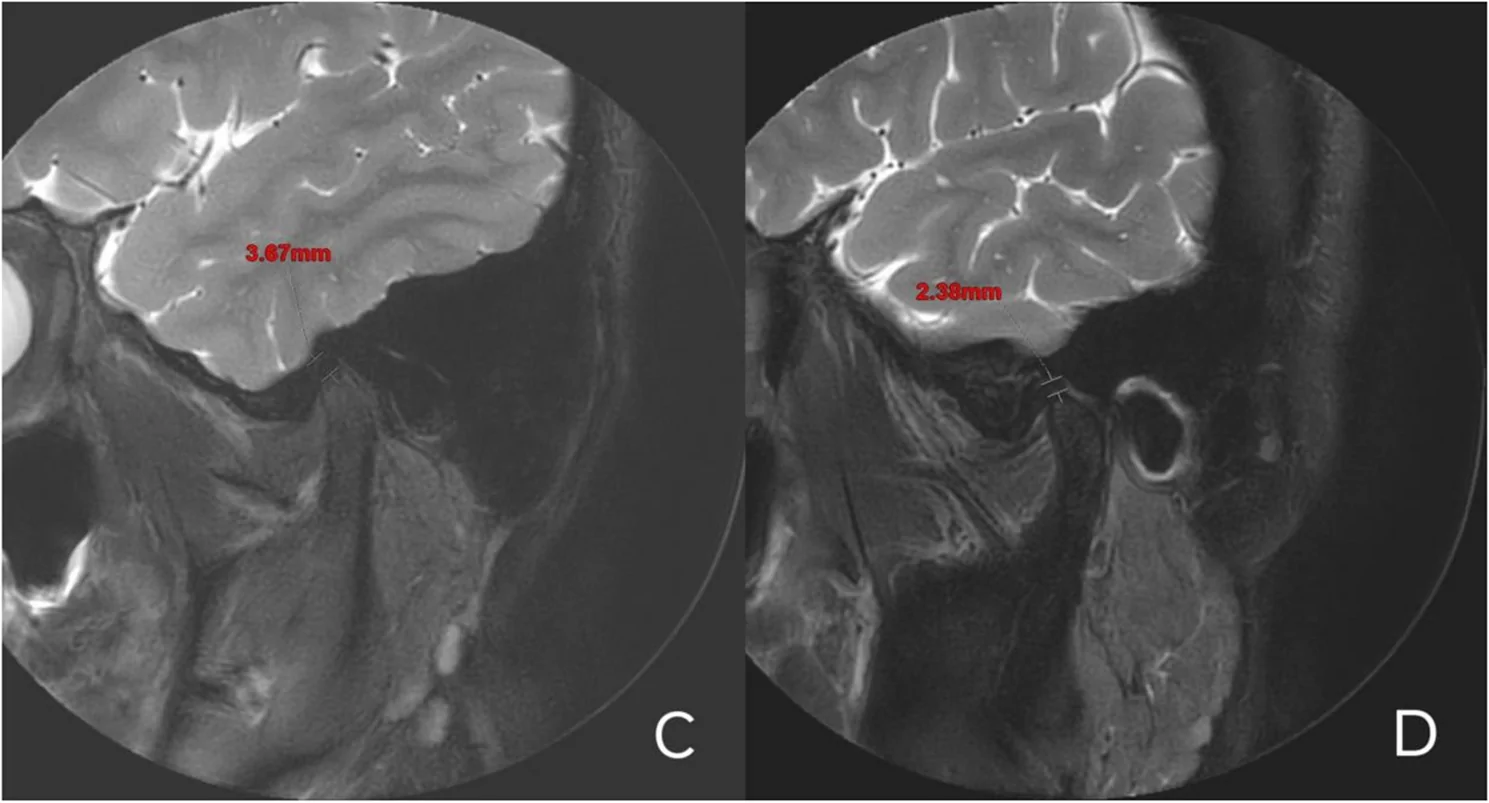
**C** Thickened posterior band (white double arrow) in an ADDwR patient; **D** normal posterior band in a comparison participant

**Fig. 3:**
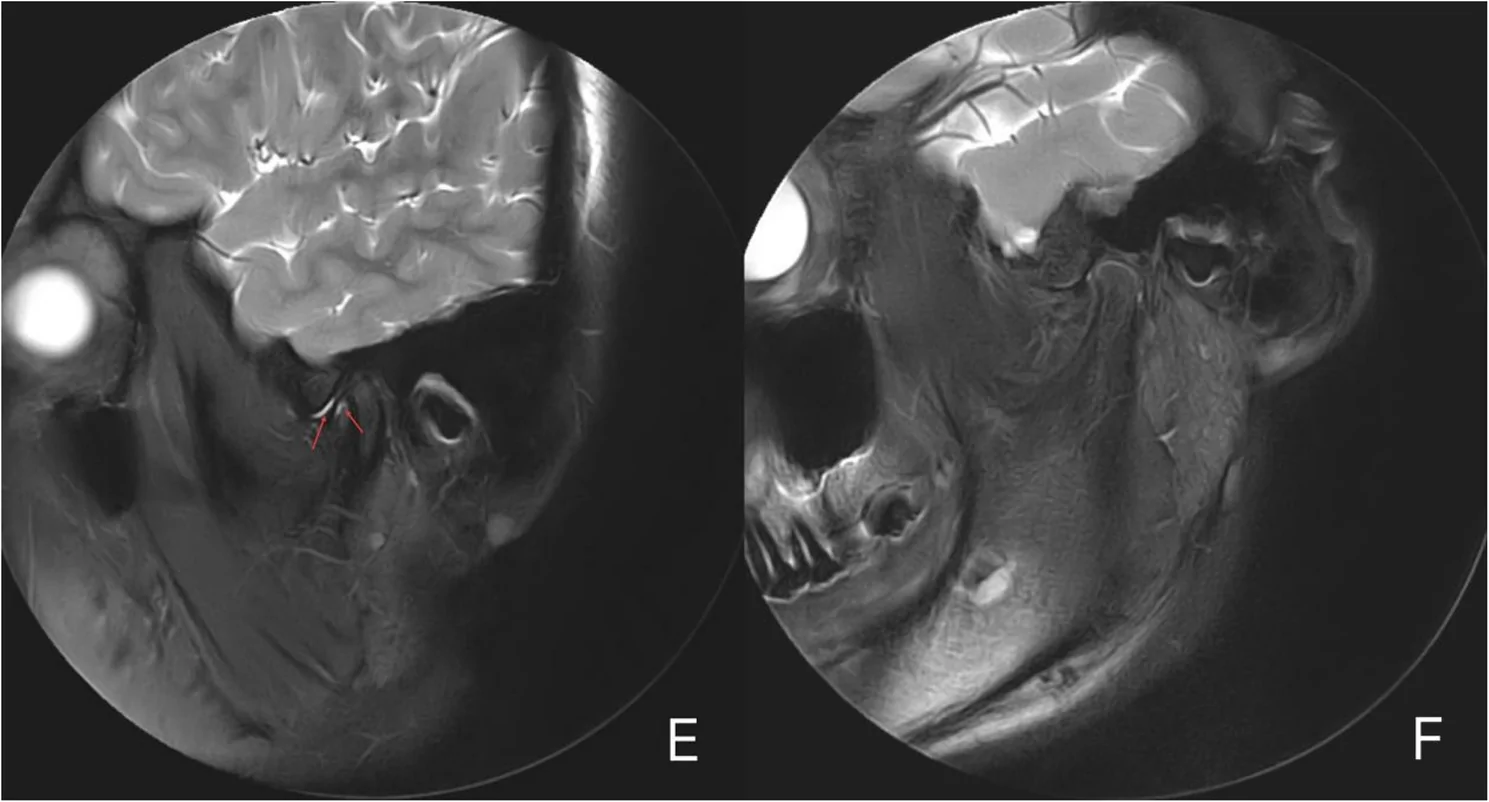
**E** Joint effusion (red arrow) in an ADDwR patient; **F** no effusion in a comparison participant

**Fig. 4:**
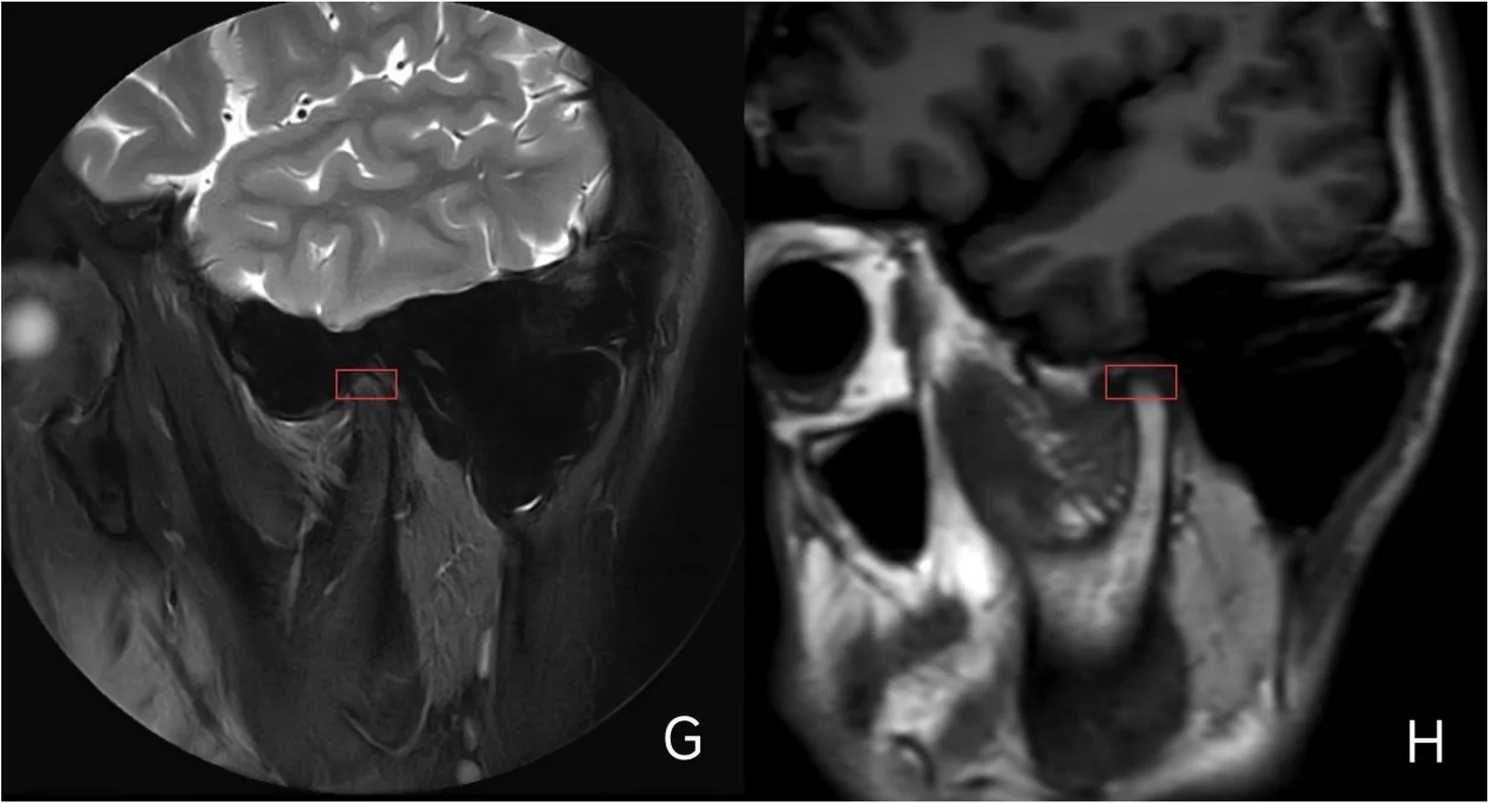
**G** Condylar BME (red box) on fat-suppressed T2WI; **H** corresponding hypointensity on T1WI

Representative MRI findings are illustrated in Figs. 1, 2, 3 and 4. In the ADDwR group, ADDwR (Fig. 1), disc deformation (posterior band thickening) (Fig. 2), joint effusion (Fig. 3), and condylar BME (Fig. 4) were frequently observed. In the comparison group, no disc displacement, disc deformation, or condylar BME was observed, and only two participants (5.0%) presented with mild joint effusion.

### Quantitative comparisons and statistical results

Table 2 summarises the MRI characteristics of the two groups. Compared with the comparison group, the ADDwR group showed higher frequencies of disc deformation (65.9% vs. none of the comparison participants, *p* < 0.001), condylar BME (27.3% vs. none, *p* < 0.001), and joint effusion (38.6% vs. 5.0%, *p* < 0.001). The posterior band thickness was also greater in the ADDwR group (median [IQR]: 3.29 [2.89–3.50] mm vs. 2.69 [2.58–2.78] mm, *p* < 0.001). Cramer‘s V for disc deformation was 0.69 (large effect) and for joint effusion was 0.40 (moderate to large effect).

**Table 2:** Comparison of MRI Findings Between ADDwR and Comparison Group

Variable	ADDwR Group (*N* = 44)	Comparison Group (*N* = 40)	*P*
Disc Deformation, n (%)	29 (65.9%)	0 (0.0%)	<0.001^&^
Average Posterior Band Thickness (mm)	3.29 (2.89, 3.50)	2.69 (2.58, 2.78)	<0.001^#^
Bone Marrow Edema of Condyle, n (%)	12 (27.3%)	0 (0%)	<0.001^&^
Joint Effusion, n (%)	17 (38.6%)	2 (5.0%)	<0.001^*^

Note: ^#^Mann-Whitney U test; ^*^Chi-square test; ^&^Fisher’s exact test. Data are median (IQR) for posterior band thickness and n (%) for categorical variables. Posterior band thickness was non‑normally distributed (Shapiro-Wilk *p* < 0.05). The comparison group was highly selected (headache patients with no clinical or MRI signs); thus, zero events do not reflect population prevalence

### Inter- and intra-observer agreement

Agreement for categorical variables (Cohen’s kappa) is shown in Table 3, and inter‑ and intra‑observer reliability for posterior band thickness are also presented. Cohen’s kappa ranged from 0.77 to 0.81, indicating substantial to almost perfect agreement. For posterior band thickness, the inter‑rater ICC was 0.95 (95% CI: 0.91–0.97), and the intra‑rater ICC was 0.96 (95% CI: 0.90–0.98), both indicating excellent reliability.

**Table 3:** Inter-observer Agreement for MRI Assessments

MRI Feature	Category	Cohen’s Kappa (κ)	95% CI	ICC(2,1)	95% CI
Disc Position	Normal/ADDwR	0.81	0.62–0.95	-	-
Disc Morphology	Normal/Deformed	0.80	0.60–0.95	-	-
Bone Marrow Edema	Normal/Edema	0.78	0.56–0.95	-	-
Joint Effusion	Absent/Present	0.77	0.55–0.94	-	-
Posterior Band Thickness (inter-rater)	Continuous (mm)	-	-	0.95	0.91–0.97
Posterior Band Thickness (intra-rater)	Continuous (mm)	-	-	0.96	0.90–0.98

Note: Kappa values were interpreted as: <0.20 slight, 0.21–0.40 fair, 0.41–0.60 moderate, 0.61–0.80 substantial, > 0.81 almost perfect agreement. *ADDwR* anterior disc displacement with reduction, *CI* confidence interval, *ICC* intraclass correlation coefficient

### Association between posterior band thickness and condylar BME

**Fig. 5:**
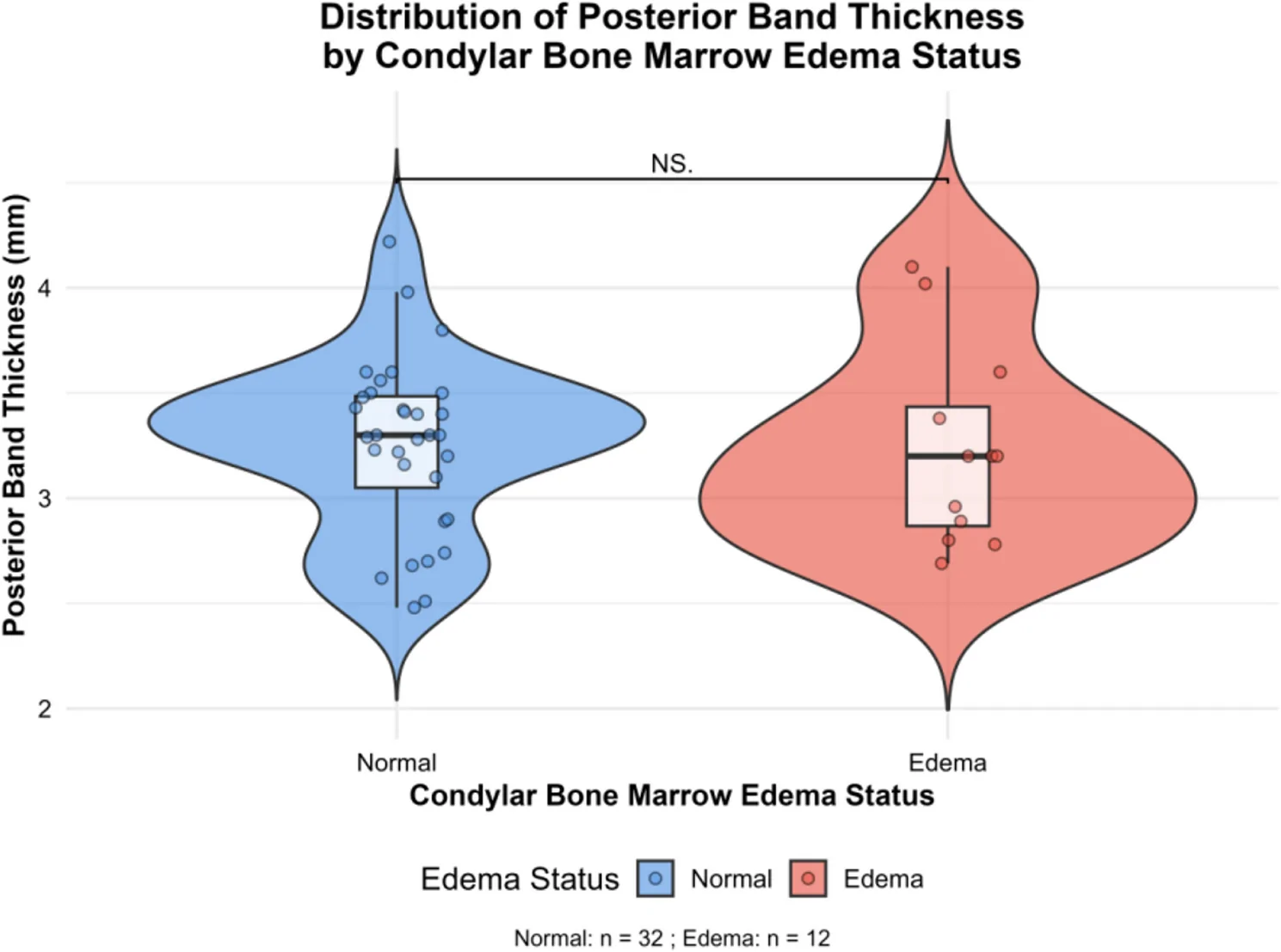
Violin plot showing posterior band thickness in ADDwR patients with and without condylar BME. Patients with BME had greater thickness (Mann‑Whitney U test, *r* = 0.44, *p* < 0.001)

To test the hypothesis that posterior band thickening is associated with BME, posterior band thickness was compared between BME-positive and BME-negative patients. In the ADDwR group, patients with condylar BME had greater posterior band thickness than those without BME (median [IQR]: 3.70 [3.45–3.92] mm vs. 3.20 [2.84–3.41] mm; Mann-Whitney U = 276.5, *p* < 0.001; rank-biserial correlation *r* = 0.44). The violin plot (Fig. 5) visually confirms this difference.

### Multivariable firth logistic regression

**Fig. 6:**
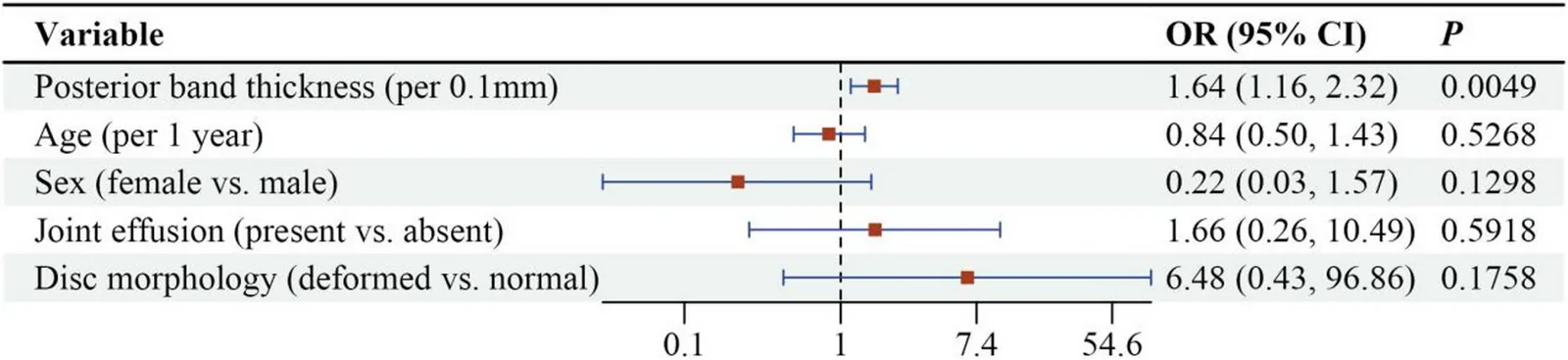
Forest plot showing adjusted odds ratios for factors associated with condylar BME (Firth logistic regression). The vertical dashed line indicates OR = 1. Squares represent point estimates, and horizontal lines represent 95% confidence intervals. The X‑axis is presented on a logarithmic scale. Detailed numerical results are provided in Table 4

Table 4; Fig. 6 display results of multivariable Firth logistic regression analysis. After age, sex, disc morphology, and joint effusion adjustment, only posterior band thickness reached statistical significance (adjusted OR per 0.1 mm increase = 1.64, 95% CI: 1.16–2.32, *p* = 0.0049). All other covariates were not significantly independently associated (all *p* > 0.05). Likelihood ratio test of overall model was significant (χ² = 42.31, df = 5, *p* < 0.001). The AIC was 88.6. The findings are exploratory as the events-per-variable (EPV) ratio was low (2.4) and need validation in larger prospective cohorts.

**Table 4:** Multivariable Firth logistic regression analysis of factors associated with condylar BME (patient-level, *n* = 84)

Variable	Coefficient(B)	SE	OR	95%CI	*P*
Posterior band thickness (per 0.1 mm)	0.495	0.176	1.64	1.16–2.32	0.0049
Age (per 1 year)	−0.170	0.268	0.84	0.50–1.43	0.5268
Sex (female vs. male)	−1.532	1.011	0.22	0.03–1.57	0.1298
Joint effusion (present vs. absent)	0.505	0.941	1.66	0.26–10.49	0.5918
Disc morphology (deformed vs. normal)	1.868	1.380	6.48	0.43–96.86	0.1758

Note: Likelihood ratio test for overall model: χ² = 42.31, df = 5, *p* < 0.001. AIC = 88.6. *B* regression coefficient on the log-odds scale, *SE* standard error

### Sensitivity analysis for within-patient correlation

Because each patient contributed two TMJs, generalized estimating equations (GEE) were attempted to account for within‑patient correlation but failed to converge due to complete separation. As an alternative, we randomly selected one joint per patient (resulting in 72 BME-negative and 12 BME-positive joints). The median (IQR) posterior band thickness was 2.8 mm (2.7–3.2) in joints without BME and 3.5 mm (2.9–3.9) in joints with BME (Wilcoxon rank-sum test: W = 242, *p* = 0.01). This alternative patient‑level selection (using a different random seed) confirmed the robustness of the association. This supports the primary finding that increased posterior band thickness is associated with condylar BME.

## Discussion

In this MRI study of adolescents with ADDwR, posterior band thickness was independently associated with condylar BME after adjusting for potential confounders. These results are consistent with previous findings showing that ADDwR is accompanied by morphological changes of the articular disc, including thickening of the posterior band, which may represent adaptive hypertrophy or fibrosis [7]. These findings also support the hypothesis that abnormal biomechanical loading is associated with posterior band thickening [8].

The reproducibility of posterior band thickness measurements was excellent, with an inter‑rater ICC of 0.95 and an intra‑rater ICC of 0.96. Regression analysis showed a robust association between posterior band thickness and BME when controlled for other covariates. A thickened posterior band may have reduced elasticity and could impair stress distribution during joint movement, which could cause abnormal load concentrations on the condyle, thus leading to microtrauma and BME [9]. The Firth regression model showed that the adjusted odds ratio for posterior band thickness was 1.64 per 0.1 mm increase (95% CI: 1.16–2.32), providing quantitative evidence for this association. However, because of the cross-sectional design, predictive value cannot be inferred.

MRI reports of the comparison group showed no disc deformities and no condylar BME, suggesting that these findings are unlikely to be incidental or normal variants in adolescents. A previous report described small physiological effusions in asymptomatic adolescents [10]; in our control group, only two participants (5.0%) had mild joint effusion, which was substantially lower than in the ADDwR group, supporting the association of joint effusion with TMD [11].

The focus on adolescents is important because the growth potential and tissue plasticity of the adolescent TMJ may lead to response patterns to disc displacement that differ from those in adults [12]. The observed increase in posterior band thickness may represent adaptive structural remodeling rather than degenerative thinning [13]. Our findings, in agreement with previous studies, suggest that posterior band morphology in TMDs evolves dynamically [14, 15]: in the early stage, it thickens in a compensatory manner and is observed together with BME, which appears to be related mainly to biomechanical stress. In later stages, prolonged pathological alterations are associated with atrophy and thinning of the posterior band [16]. In that context, BME is more closely linked to the inflammatory processes of secondary osteoarthritis [17]. To our knowledge, this study provides new information on this early compensatory phase in adolescents.

The present study extends the discussion on TMD beyond the simple description of disc-condyle relationships to the relationships between morphology, function, and early degeneration. The high prevalence of disc deformation in ADDwR patients is consistent with many studies that observed disc morphological changes as a typical trait of TMD [18, 19]. Özel et al. [3] described how certain disc deformation patterns correlate with clinical clicking, indicating that morphology is involved in joint function. This study has advanced the understanding by not only observing morphological changes but also quantifying posterior band thickness and identifying it as an independent correlate of BME. These findings support the hypothesis that abnormalities in disc morphology may serve as biomechanical markers of abnormal joint load distribution and also suggest that posterior band thickening may be due to adaptive remodeling but could also be associated with subsequent degenerative changes [20, 21].

In terms of condylar changes, BME is the focus of this study, which is a departure from the classical phenomena of bone erosion or sclerosis. Various studies have shown that bone erosion [22, 23] and subchondral bone remodeling [24] are radiological indicators of the late or definitive stage of TMJ OA which usually means irreversible structural defects. In contrast, BME is believed to be an early active stage of OA which may still be reversible [25]. The manifestations seen on MRI are thought to result from active pathophysiological processes, such as vascular congestion, tissue edema, and infiltration of inflammatory cells in the bone marrow cavity, induced by abnormal mechanical or inflammatory stimuli [26].

Therefore, these findings suggest that BME may be an early radiologically detectable finding in the progression from disc dysfunction to structural OA. This provides a theoretical foundation for recognizing a potential time window for clinical interventions before irreversible damage occurs, although prospective studies are needed to confirm this temporal relationship.

The findings suggest that thickening of the posterior band is associated with condylar BME, implying a possible continuous degenerative process, and that abnormal joint biomechanics may be an underlying mechanism [27]. Thickening and deformation of the articular disc, especially the posterior band, may alter its stress-absorbing and distributing functions during anterior sliding over the condyle [28]. Finite element analyses have suggested that changes in disc shape are associated with abnormal concentration of stresses transmitted to the condylar surface, particularly posterolaterally [29]. Long-term abnormal mechanical loading has been hypothesized to trigger cellular and molecular reactions in subchondral bone, including micro-fractures of trabecular structures, increased vascular permeability, and exudation of inflammatory mediators [30, 31]. These changes together may manifest as BME signals on MRI. Accordingly, this study provides imaging evidence supporting the existence of such a pathological process in adolescents with ADDwR, with posterior band thickening as a quantifiable imaging feature associated with BME.

These findings, if validated in future prospective studies, could inform the development of MRI-based imaging biomarkers for the assessment of adolescent ADDwR. However, clinical applications such as risk stratification or early intervention cannot be recommended based on this single retrospective cross-sectional study.

Strengths of this study include the use of standardized clinical criteria (DC/TMD), frequency matching for age and sex, high inter-observer agreement, and the application of Firth logistic regression to address rare events. Nevertheless, several limitations should be emphasized. First, owing to the cross‑sectional design, causal inferences cannot be drawn. The observed association does not imply directionality, and alternative explanations—such as reverse causation (e.g., BME influencing disc morphology) or residual confounding (e.g., occlusal issues, parafunctional habits, hormonal status)—cannot be excluded. Second, the sample size was modest (84 participants, with only 12 BME events), resulting in a low EPV ratio (2.4), which limits statistical power and model stability. Formal model diagnostics (e.g., goodness‑of‑fit tests, multicollinearity assessment) were not performed owing to the small sample size and the exploratory nature of the study; any such analysis would have low statistical power and could be misleading. Future studies with larger samples should include these assessments. A notable statistical observation is that the overall likelihood ratio test for the model was highly significant (χ² = 42.31, df = 5, *p* ≈ 5 × 10⁻⁸), yet only posterior band thickness was individually significant among the five covariates. This pattern is likely driven by the strong association of posterior band thickness with BME, combined with complete separation (zero events in the control group for BME and disc deformation). The other covariates, while contributing to the model fit collectively, lack sufficient statistical power (due to the low EPV of 2.4) to reach individual significance. This discrepancy does not indicate model misspecification but rather reflects the dominant role of the key predictor in a small, rare-events dataset. Third, as a single‑center study, the generalizability of the findings to other populations or imaging protocols is uncertain. Fourth, the comparison group was derived from a hospital‑based sample (headache patients) rather than from healthy community volunteers, which may introduce selection bias, although all comparison subjects underwent thorough clinical and MRI screening to rule out TMD. Additionally, potential undiagnosed factors such as bruxism in the comparison group were not investigated, which may have influenced the results. Fifth, static MRI does not provide information on dynamic functional biomechanics; future studies should consider dynamic imaging techniques. Sixth, the use of binary classification for complex morphological variables (e.g., disc deformation) simplifies continuous or ordinal features, which may result in information loss and misclassification. This was a pragmatic choice given the small sample size, but it remains a limitation. Seventh, potential confirmation bias is also acknowledged, as the interpretation primarily focused on the hypothesized pathway. Eighth, several potential confounders—such as symptom duration, bruxism, trauma history, and occlusal factors—were not available in this retrospective dataset. In summary, the results should be regarded as hypothesis‑generating rather than conclusive and require validation in larger, prospective, multicenter studies.

Future prospective longitudinal studies with larger samples are required to determine whether posterior band thickening and other MRI features could act as prognostic biomarkers for BME progressing to end‑stage OA. Also, dynamic or quantitative MRI imaging techniques (for example, T2 mapping [32]) might yield further information on early biochemical changes before any morphological changes become apparent.

## Conclusion

In this retrospective cross-sectional study of adolescents with ADDwR, posterior band thickness was independently associated with condylar BME. These findings suggest that, although morphological changes in the articular disc and early bone edema may be detectable by MRI in adolescence, it is not possible to infer causal relationships. Therefore, the results should be considered hypothesis-generating. Future prospective studies are needed to determine whether these imaging features can predict long-term outcomes or inform clinical management strategies.

## Supplementary Information

Supplementary Material 1.

## Data Availability

The datasets generated and analyzed during the current study are not publicy available due to privacy restrictions but are available from the corresponding author on reasonable request.

## Abbreviations

ADDwR: Anterior disc displacement with reduction
BME: Bone marrow edema
MRI: Magnetic resonance imaging
OA: Osteoarthritis
PDW: Proton density-weighted
TMD: Temporomandibular disorders
TMJ: Temporomandibular joint
T1WI: T1-weighted
T2WI: T2-weighted
TSE: Turbo spin echo
VAS: Visual analog scale
