# Supplementary material for: The relationship between disc morphology and condylar bone marrow edema in adolescent patients with anterior disc displacement with reduction: a retrospective cross-sectional study
Source: BMC Oral Health. 2026 Jun 12;26:1597. doi: 10.1186/s12903-026-08827-0 (PMC13508436; doi:10.1186/s12903-026-08827-0)
Supplement: Supplementary file 1 [file 12903_2026_8827_MOESM1_ESM.pdf]

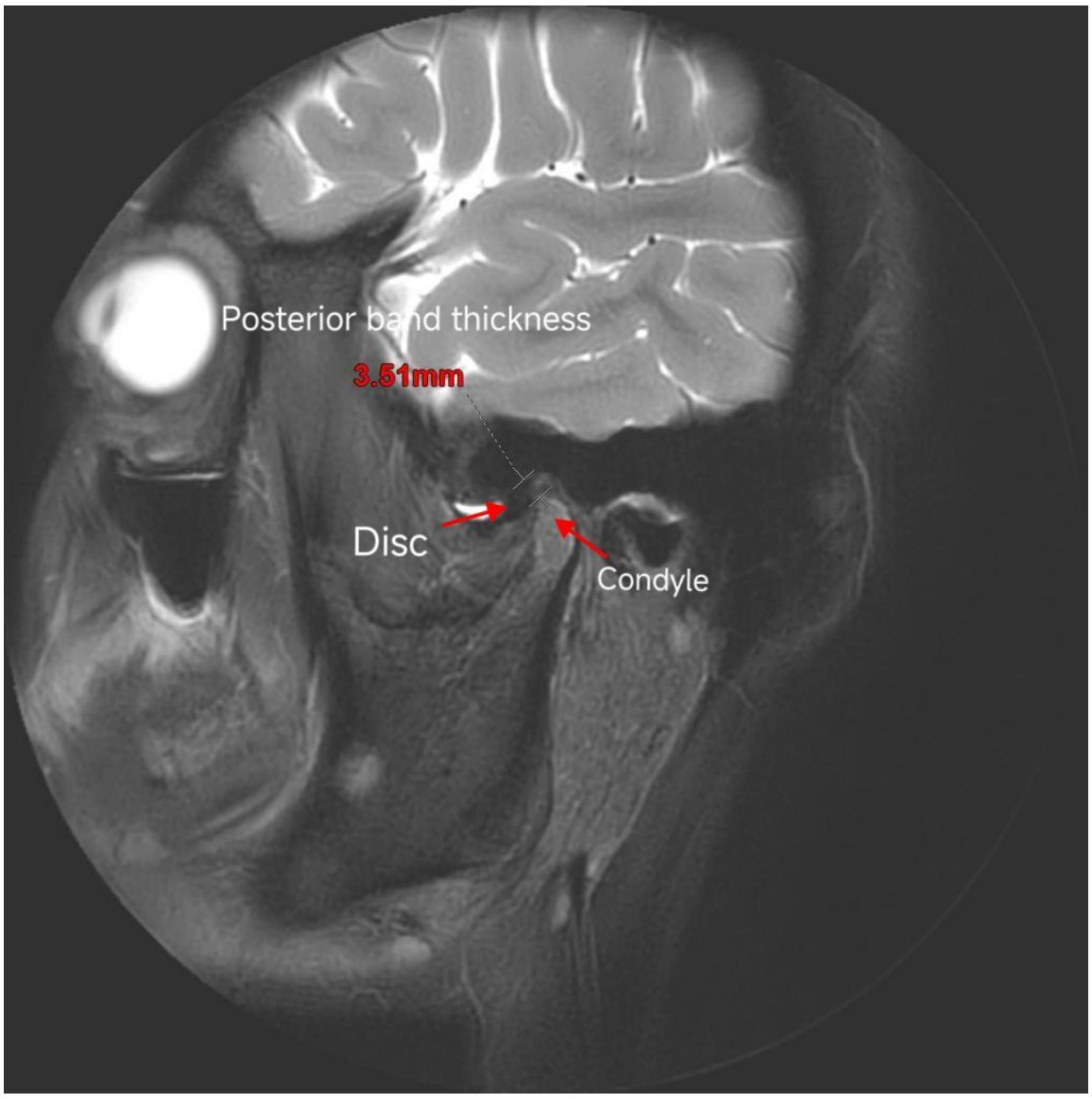

Fig. S1 Schematic diagram of posterior band thickness measurement.

This image was obtained from a 14-year-old patient with ADDwR (right TMJ, oblique sagittal T2-weighted fat-suppressed image, closed-mouth position). The condyle and the posterior band of the disc are labelled. The white double-headed arrow indicates the measurement of posterior band thickness: on the slice with the clearest disc profile, the maximum distance from the superior to the inferior border of the posterior band is measured perpendicular to its long axis. Measurements were performed using PACS-integrated callipers (precision 0.01 mm), and three independent measurements per joint were averaged.
